# Is pathogen prediction possible?

**DOI:** 10.1371/journal.pbio.3003162

**Published:** 2025-05-15

**Authors:** Arturo Casadevall

**Affiliations:** Department of Molecular Microbiology and Immunology, Johns Hopkins School of Public Health, Baltimore, Maryland, United States of America

## Abstract

To what extent can we predict future pathogenic microbes? This Perspective discusses why complex requirements for virulence and dynamic host-microbe interactions make probabilistic predictions difficult, but not impossible.

## Teaser

As humanity comes into contact with new microbes, there is a need to identify which might be future pathogenic threats. Host–microbe interactions manifest emergent properties and chaotic dynamics, posing limits on prediction. However, probabilistic predictions are possible.

Around 1980 I was taught in medical school that retroviruses were not pathogenic for humans and that coronaviruses were at most a winter nuisance causing colds and rhinorrhea. That statement was based on what was known about the causes of infectious diseases at the time. Within a year the retrovirus Human T-lymphotropic Virus 1 was reported to cause adult T cell leukemia [1], and the first cases of the HIV pandemic would be described, later shown to be caused by Human Immunodeficiency Virus (HIV), also a retrovirus [2]. Two decades later, three coronaviruses (SARS-CoV, MERS-Cov, and SARS-CoV-2) would cause deadly outbreaks and a pandemic in the first decades of the 21st century [3]. Clearly, history and experience with infectious diseases do not predict future threats, thus raising the fundamental question of whether prediction is possible for future pathogenic microbes.

Before considering prediction, it is worthwhile to take stock on what we know about pathogenic microbes. Despite millions of viruses, prokaryotic, and eukaryotic microscopic species, only a small minority of this microbial set is pathogenic for animals. In 2021, it was estimated that there were 1,521 bacterial species that were pathogenic for humans [4]; while these numbers are almost certainly an underestimate, we are dealing with a very small number considering estimates of 1 billion species worldwide [5]. For viruses, there are 10–100 pathogenic species per mammal from an estimated 10^7^ to 10^9^ viral species [6]. While such numbers are highly uncertain and almost certainly underestimates, the point is that the proportion of microbes with the capacity to cause disease in any human is very small. This in turn implies that the characteristics needed for virulence are rare and/or that humans have formidable immune defenses that can prevent the overwhelming majority of Terran microbes from establishing themselves in the host.

**Fig 1:**
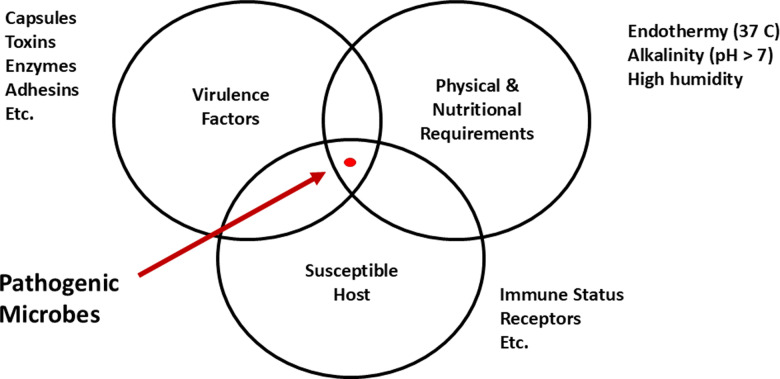
Requirements for microbes to survive in a human host. The relative scarcity of pathogenic microbes reflects the fact that only a minority have susceptible hosts and both the necessary virulence factors and physiological characteristics that allow survival, persistence, and replication in the host.

Given that the capacity for virulence among microbial species is rare, it is worthwhile considering the minimal requirements for microbial survival in a host. Since survival in a host is generally a requirement for virulence, then it follows that traits that confer property are also needed for virulence. Following this logic, I identify three general categories that must be met by a microbe to be pathogenic: (1) The ability to survive within the physical (e.g., temperature) and chemical constraints imposed by the host environment; (2) possession of attributes (virulence factors) that allow it to establish itself in the host and resist immune eradication; and (3) host susceptibility, which includes immunological history, genetics, the presence of necessary receptors for attachment and cellular internalization, etc. (Fig 1). Meeting these criteria is not enough, however, since for a microbe to cause disease it must damage the host sufficiently to impair homeostasis and that damage can come from the microbe, the host immune response, or both [7]. These complex requirements likely explain why there are so few pathogenic microbes. In fact, elimination of a single virulence factor, such as a capsule protein or toxin, is often sufficient to render a pathogenic microbe avirulent [7]. In other words, the capacity for virulence is such a demanding phenotype that it is rare and can be fleeting.

Much is known about how microorganisms can become pathogenic. The emergence of immunocompromised hosts showed how impairment of immunity could lead microbes previously thought to be nonpathogenic to cause disease. For example*, Candida albicans* went from rarely causing disease to a major pathogenic fungus during the 20th century, and transitioned from commensal to pathogen without genetic changes. The emergence of HIV [8] and highly pathogenic coronaviruses [3] showed how animal viruses can rapidly adapt to human hosts through acquisition of mutations that facilitate replication and disease. Meanwhile, most fungi cannot tolerate mammalian temperatures but some could acquire tolerance of higher temperatures from adaptation to global warming and this could explain the recent emergence of *Candida auris* as a major human fungal pathogen [9]. In considering future threats, the analysis of recent new pathogenic microbes can provide information for prediction. For example, one can anticipate that a virus from bats, a group of species that shares the mammalian class and body temperature with humans (while not hibernating), is more likely to pose a threat than a salamander virus, with the caveat that simple rules will not be sufficient to prioritize threats among thousands of bat viruses. Similarly, an insect pathogenic fungus that cannot survive above ambient temperature is less of a threat to humans than one adapted to growing at near human temperatures. Such considerations contribute to zoonotic risk assessments [10]. Hence, dissection of mechanisms of human adaptation and the history of infectious diseases, combined with increased surveillance of animal diseases, can help to prioritize threats. In fact, there are ongoing efforts to identify potential zoonotic viruses based on generating predictive models that combine genomic data with history and network analysis, etc. that could identify the highest threats to humans [11]. Prediction may be more feasible for viruses than for prokaryotic and eukaryotic pathogenic microbes given the relative genomic simplicity of the former, which makes the identification of critical facets of the host–microbe interaction such as virus receptors and evasion strategies easier to model.

Apart from the epistemic limits imposed by emergence, there is additional uncertainty in whether prediction is possible when one considers the dynamics of the host-microbe interaction. Any predictive scheme would require some ability to predict outcomes from such interactions, including the magnitude and efficacy of immune response, which in turn depend on host genetics and the immunological history of the individual, as prior exposures to other or the same microbe would affect susceptibility by influencing the quantitative and qualitative aspects of the immune response. Unfortunately, relatively little work has been done to understand the dynamics of host-microbe relationships. A recent study found evidence for chaotic dynamics in *Pseudomonas* spp.-infected worms and flies [12]. Chaotic systems are deterministic, but since they have outcomes that are highly sensitive to the initial conditions, they are essentially unpredictable. The initial conditions for host-microbe interactions include a myriad of variables such as inoculum, immunological state of the host, temperature, humidity, and host–microbiome composition, and if the system follows chaotic dynamics, tiny changes in any of these conditions can produce unpredictable outcomes.

However, probabilistic predictions may be possible. Consider weather, a chaotic system for which there have been consistent improvements in forecasting over the past century. Improvements in weather forecasting have driven improvements in observation (e.g., satellites), more sophisticated understanding of atmospheric physics, more accurate models, and faster computers [13]. Perhaps the field of microbial pathogenesis can take a page from the science of weather prediction and combine increased sampling of the natural world with clinical observations, experimental results of pathogenesis experiments, and historical data to identify potential threats and assign a risk probability. However, the success of such predictive models will require learning how to integrate such different types of data with host susceptibility and evolving immune responses. Although this type of predictive analysis is currently beyond the analytical horizon in the biomedical sciences, it is noteworthy that the field of microbial pathogenesis has an enormous potential advantage over weather scientists in that it can do experiments to test virulence predictions.

In summary, even if certainty in prediction is not possible in microbial pathogenesis, it may be possible to construct a probabilistic framework to identify those microbes that pose the greatest threats. A form of prediction that incorporates history and antigenic characteristics is practiced each year in selecting strains for the yearly influenza vaccine [14], which although not perfect, provides both an example and a precedent for the feasibility of predictive schemes in infectious diseases. In fact, developing predictive models for the identification of future threats and testing predictions in animal models could supercharge progress in the field by allowing the identification of relevant variables and their relative importance.

## Abbreviation:

HIV: Human Immunodeficiency Virus
